# Investigating polyurethane foam loaded with high-z nanoparticles for gamma radiation shielding compared to Monte Carlo simulations

**DOI:** 10.1038/s41598-024-67031-8

**Published:** 2024-07-15

**Authors:** Mahdieh Mokhtari Dorostkar, Haleh Kangarlou, Akbar Abdi Saray

**Affiliations:** 1 Department of Physics, Urmia University, Urmia, Iran; 2 Department of Physics, Urmia Branch, Islamic Azad University, Urmia, Iran

**Keywords:** Radiation shield, Polymer, Nanocomposite, Gamma ray, MCNPX, Physics, Applied physics, Nuclear physics

## Abstract

Since the beginning of research into radiation and protection against it, the importance of searching for proper materials against radiation hazards has been recognized. Gamma radiation protection materials usually deal with heavy elements with high prices, which are hard to maintain. Polyurethane-based (PU) materials are popular in sound and thermal insulation due to, their low-weight properties and, most importantly, fast and convenient construction ingredients. PU foams (PUF) can be used as radiation shield and noise and heat resistance due to their approachability, light-weight, high resistance, and comfortable construction. This study involved simulation and an experiment to construct and investigate the properties of Polyurethane material doped with lead oxide as a gamma shield. The shield was considered in several weight fractions of lead, yielding several samples. The MCNPX 2.6 Monte Carlo code has been utilized for simulation procedure, and ^137^Cs was employed as the gamma source in both simulation and experiment. The results offer a promising response against the gamma radiation and are suitable for attenuating gamma rays.

## Introduction

Appropriate shielding material selection has always been challenging in radiation shielding studies. It is important to provide safe and secure environments in different radiation applications, such as medical facilities and research reactors. Therefore, it is necessary to study and develop radiation shielding material^1,2^. Studies based on composite materials with different filler candidates have recently been conducted. Polymer composite shielding materials are lighter, cheaper, flexible, durable and therefore, a proper gamma shielding material. In addition, polymer composites have some advantages over metals, including their workability, flexibility, low cost, chemical stability, and volume diminishment after use^3–5^.

Polymeric composites can be a great candidate in neutron shielding, considering their structure, which includes hydrogen and carbon atoms. Moreover, adding a small amount of high atomic number material aids gamma and X-ray shielding^6,7^.

Polyurethane foam (PUF) is a well-known material in industry. They differ in properties due to their structure and ingredients. This material is formed by the reaction of polyols and isocyanates. PUF ranges from rigid pneumatic resins to flexible porous elastomers. Rigid PUF is used primarily as an insulating material in construction, piping, and packaging. Flexible PUF is utilized as a cushioning material in furniture, bedding, carpet underlay, automobiles, and packaging^8^. In addition, PU foam is well-known as sound and thermal insulation^9^ and can be a great eco-friendly material^10^. In this study, we attempt to convert PUF to a potent shielding material.

Several studies have been dedicated to the shielding properties of PUF for electromagnetic shielding^11–13^. For example, Gosh et al.^14^ suggested PUF as a great electromagnetic shielding material. It is flexible, water durable, and highly electronic conductive. They upgraded PUF by adding Ketjen black to improve its properties.

Xiangyu Zheng et al.^15^ also suggested PUF as a light weight electromagnetic shield with high absorption. According to them, high absorption materials can reduce the secondary pollution caused by electromagnetic wave reflection. They added Fe_3_O_4_@polyvinyl alcohol (Fe_3_O_4_@PVA) and graphene oxide@silver (GO@Ag) to the PU matrix and constructed Fe_3_O_4_@PVA and GO@Ag/PU composite foam by foaming. The combination resulted in a great protection material.

Hence, PUF is a popular material in electromagnetic shielding studies. However, some studies have considered their radiation shielding properties.

For example, Franco Cataldo and Michele Prata^16^ explained some polymers, which are regarded as shielding materials. According to them, composites offer freedom in the construction of a new shield. However, more studies have investigated well-known shielding polymers like Polymethyl methacrylate (PMMA) or polypropylene (PP) rather than the wide possible range of other polymers. These studies investigated Polyurethane as a neutron shield and suggested it for neutron and other ionizing radiation. PU can be derived from renewable resources and can accept high loading of active shielding materials without disadvantages in its properties.

Some studies examined different fillers including amorphous boron and boron nitride at 21% of loading weight. The resulting material remained flexible after loading the fillers and had better shielding outcomes against thermal neutrons compared to unloaded PU.

Minxuan Ni et al.^17^ made a polyurethane shield loaded with heavy oxides and investigated the mechanical properties, cohesion strength, curing time and viscosity test. The results were promising in terms of low energy gamma protection.

Tatsuya Maeda et al.^18^ developed an elastic shield with significant X-ray shielding ability. They used Bi_2_O_3_ particles in porous polyurethane. They dipped the porous polyurethane in an aquatic solution and added metal particles. Then, the mixture was air-dried. Promising results were obtained in radiation protection for 70 kV X-rays.

Hanson, S.C. et al.^19^ studied high atomic number (Z) fillers, such as tungsten oxide, with a polyurethane coating, yielding results in X-ray protection of the 123–247 keV energy range. Moreover, the effect of gadolinium oxide infusion was studied in the mentioned energy regime.

Another example is the study by Elisa Toto et al.^20^, who explored space radiation protection. Polymer-based materials are important in space radiation protection due to their low weight and mechanical properties tailored to spacecraft components. The mentioned study described polyethylene (PE), polyimide (PI), polydimethylsiloxane (PDMS), and some other polymers and fillers involved both numerical and experimental investigation.

The present study is an effort to investigate the feasibility of using PUF as a polymeric part of a gamma shield through simulation and experiment.

## Materials and methods

### Theory

Some important shielding parameters are explained in this section. The Linear attenuation coefficient or LAC (µ(cm^-1^)) is the probability of particle interaction with a physical process per unit distance traveled. Meanwhile, the Mass Attenuation Coefficient or MAC (cm^2^g^-1^) is defined as ${\mu }_{m}=\mu /\rho$. From the Beer Lambert law^21,22^, we have:

$$I={I}_{0}{e}^{-\mu x}$$

1 $${\mu }_{m}x=\text{ln}\left(\frac{{I}_{0}}{I}\right)$$

In Eq. (1), *x* is the thickness of the sample (cm or mass thickness (g.cm^-3^)), *I*_*0*_ is the incident particle intensity, and *I* is the attenuated photon intensity^23^.

Moreover, the Half Value Layer (HVL) refers to the thickness of the matter in which the intensity of the incident beam is reduced by half. This variable depends on thickness. HVL (cm) can be calculated by Eq. (2):

2 $$HVL=\frac{ln2}{\mu }$$

Similarly, the Tenth Value Layer (TVL) is the thickness of the sample in which the incident beam is reduced by one-tenth compared to the primary place. Equation 3 represents the TVL (cm):

3 $$TVL=\frac{ln10}{\mu }$$

Some other parameters, such as Mean Free Path (MFP), the mean distance that a particle travels before stopping, transmission factor (TF) the number of particles transmitted through the sample compared to the initial intensity, and *Z*_*eff*_ can be considered in shielding problems^24^.

### Simulation

Simulation, as a research scheme, is the first step of every project in nuclear studies. There are several nuclear codes that can be used for particle transport and other purposes. The present study utilized the Monte Carlo N-Particle Transport Code System Extended (MCNPX) version 2.6.0 (Los Alamos National Lab, USA) to calculate of mass and linear attenuation coefficients. The ENDF/B-VII data library was used for particle transport and interactions with the considered media. MCNP can provide three-dimensional (3D) simulations for investigating radiation interactions with a minimum energy 0.001 MeV for photon particles. It considers different cross-section libraries and utilizes physics models for particle types^25^. Various studies investigated Nano structures with MCNP^26,27^. Although, tally measurements cannot be made on this scale, the geometry and interactions in other volumes, such as detectors are possible. In MCNP simulations with gamma source, the most significant difference between Nano-sized particles with other particles is density. So, the density of the available Lead Oxide Nanoparticles was measured with Gravimetric buoyancy method. This method is widely used for density measurements and utilizes Archimedes’ principle states that a solid immersed in fluid experience buoyant force acting upwards on it. At first, the sample was weighed with a 4-digit analytic balance in air then immersed in auxiliary liquid with known density. The density of the sample was measured from the known density of the liquid and the mass values.

**Figure 1:**
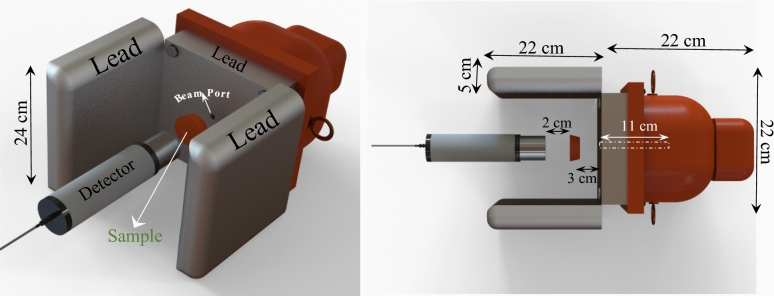
Considered geometry in MCNP simulation.

The geometry of the experiment is simulated as in Fig. 1. A truncated cone was used as a sample, in which the radius of the base and top and the height equaled 2.75 cm, 2.5 cm, and 2 cm respectively. A 22 × 22 × 22 cm lead cube was considered as the source shield in which the source was located in the middle, and the beam was guided with a beam tube of 0.5 cm radius through the sample. The NaI (Tl) detector (radius of 2.54 cm and height of 5.08 cm) and the sample were between two lead walls of 22 × 24 × 5 (Fig. 1).

**Figure 2:**
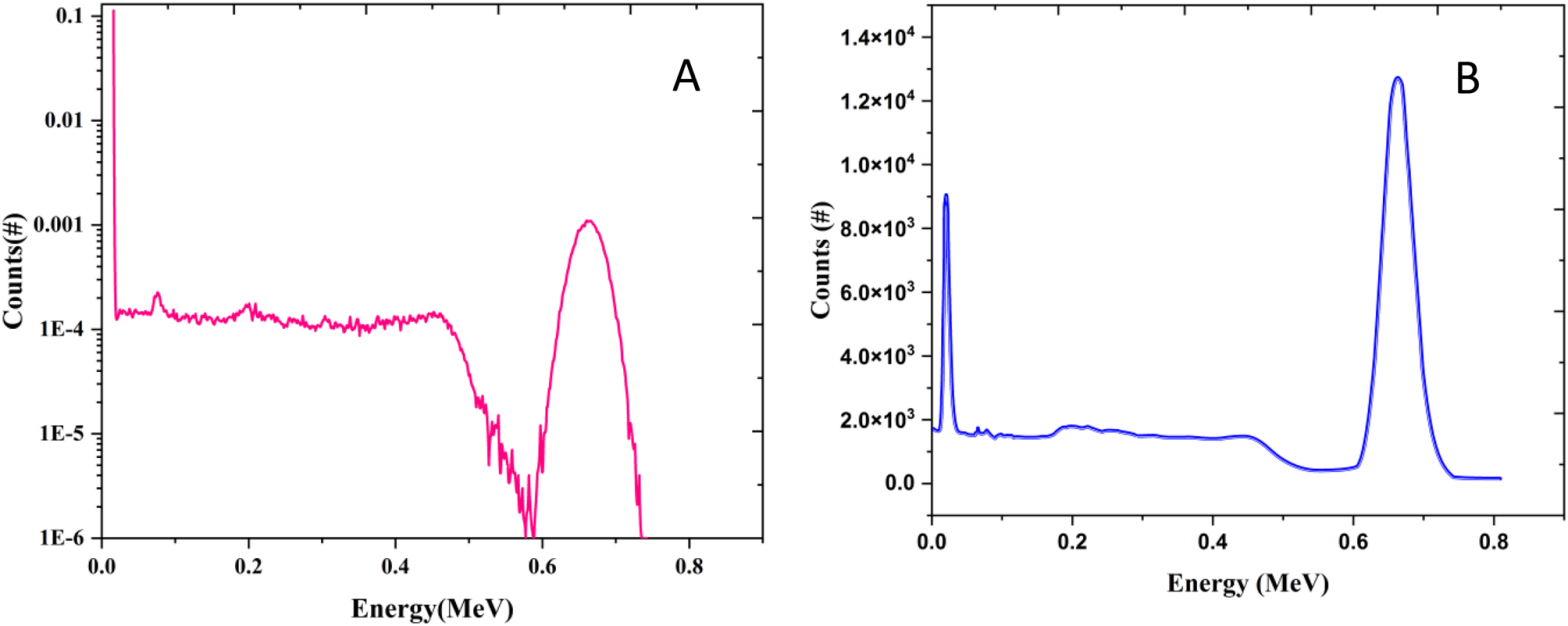
137Cs spectrum.

A ^137^Cs (20 mCi-1985) was used as the point source. The sample was located in 3 cm away from the beam port, and the detector had 2 cm distance from the sample. The gamma spectrum of the source was simulated with tally F8 and Gaussian energy broadening (GEB) to have a better view on 662 keV energy peak. Figure 2 is reporting source spectrum in simulation and experiment. Panel A is the simulated spectrum in the MCNPX and B is the experimental spectrum. The differences between spectrums are because of GEB card, tally F8 and per particle results in MCNP. However, both spectrums are showing the 662 keV energy peak, Compton edge and backscatter peak obviously and in same energy.

The expected particle counting in the simulation was selected as a photon (par = 2) for variance reduction, to reduce statistical error, and to optimize the computer processor by ignoring the unused particle counting for output data. The STOP command was used for error of tally F4 and the code stopped when the error was less than 1%.

Gamma particle count in the entrance surface of the detector and in the detector’s volume was calculated through this geometry. Tally F1 and F4 calculated the counts in the detector iterance window and flux in the detector volume. MCNPX calculations were completed by using Intel^®^ Core ™ i7 CPU 3.6 GHz computer hardware.

### Experiment

Lead oxide nanoparticles were generated mechanically method. A ball mill machine was used for this purpose. A ball mill is usually a rotating container machine containing some grinding balls. When the container rotates, the grinder balls move, which reduces particle size. In fact, this process works on the principle of impact and attrition. This size reduction is the result of dropping balls from near the top of the shell^26^.

**Figure 3:**
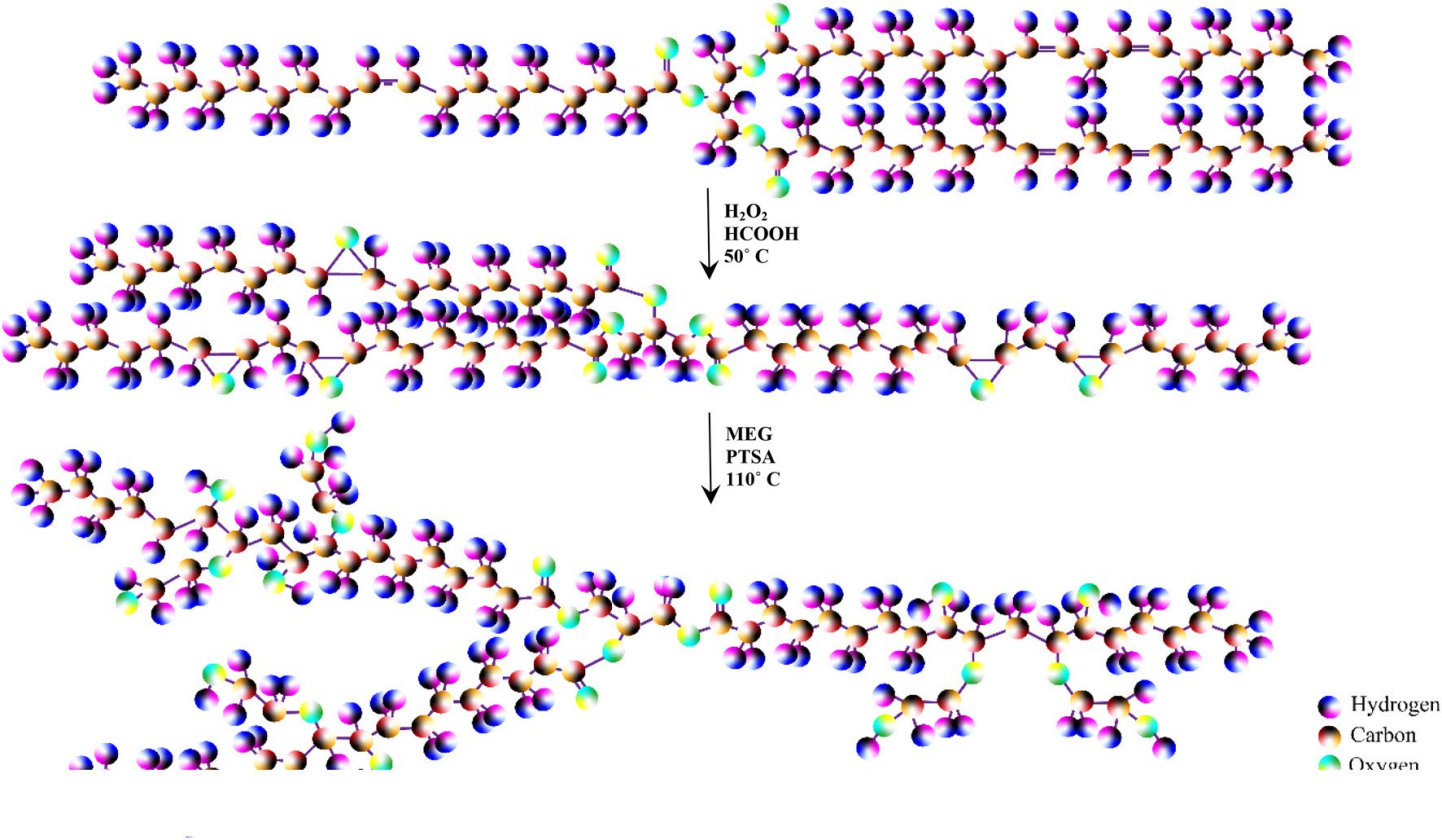
Reactions.

The reaction of polyol synthesis schemes is presented in Fig. 3. The two-step method involved ESFO (epoxidation of the double bonds of sunflower oil) and the reaction of opening the oxirane rings using ethylene glycol. In addition to traditional caster oils, epoxidation of the double bond, and eventually, the ring opening of epoxide groups are the most important reactions to introduce the hydroxyl groups into the vegetable oil’s structure^27,28^.

In order to synthesis the desired polyurethane, the polyol component was reacted directly with the isoyol component via a one-step procedure.

**Figure 4:**
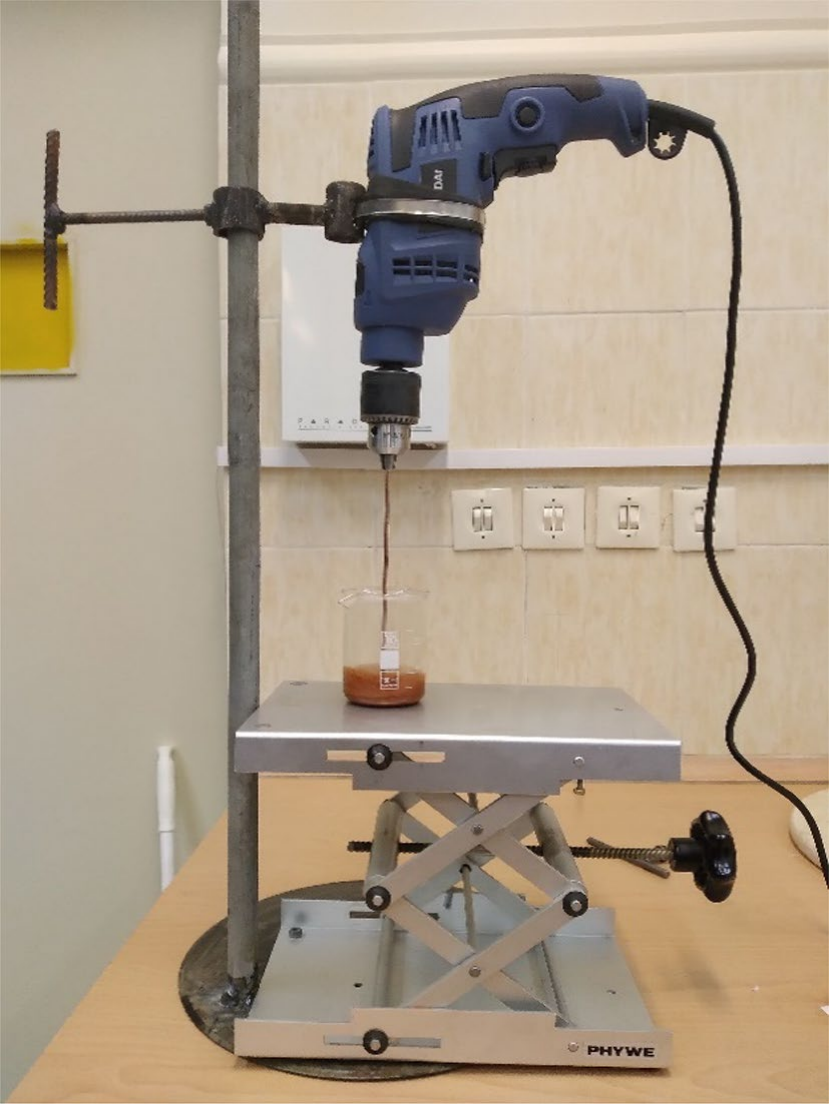
Mixing process of Polyol and Isoyol.

The synthetic procedure was carried out based the following description: The polyol and high-Z nanoparticles were added to the stirrer and mixed for several minutes to have a uniform mixture (see Fig. 4). Then, they were poured into an open mold. It is better to use a container so that the foam can be removed easily. The isoyol was utilized for the foam system to obtain non-collapsing and stable foams. Foaming took place by adding isoyol and stirring for a few seconds. Then, the temperature of the container was increased, and free rise foaming took place in the vertical direction. The whole foaming process happened in a few seconds, and then the foam became firm and strong. If the sample wasn’t taken out of the stirrer in a few seconds, it would be stocked. To solve this problem a drill was deemed the best option for mixing.

Table 1 reports the properties of the generated samples, including the weight fraction of each element in the samples and their density (gr.cm^−3^).

**Table 1:** Generated samples properties.

PU	PbO	C	H	N	O	Pb	Density
0.998	0.002	0.590058	0.065562	0.050993	0.29153	0.000735	0.96172
0.995	0.005	0.588285	0.065365	0.050839	0.290654	0.001839	0.964311
0.99	0.01	0.585328	0.065036	0.050584	0.289195	0.003677	0.96866
0.96	0.04	0.567591	0.063066	0.049051	0.280435	0.014709	0.995603

After constructing the foam with lead oxide as an additive for gamma radiation shielding properties, the obtained shielding material was located in front of the experimental set-up.

**Figure 5:**
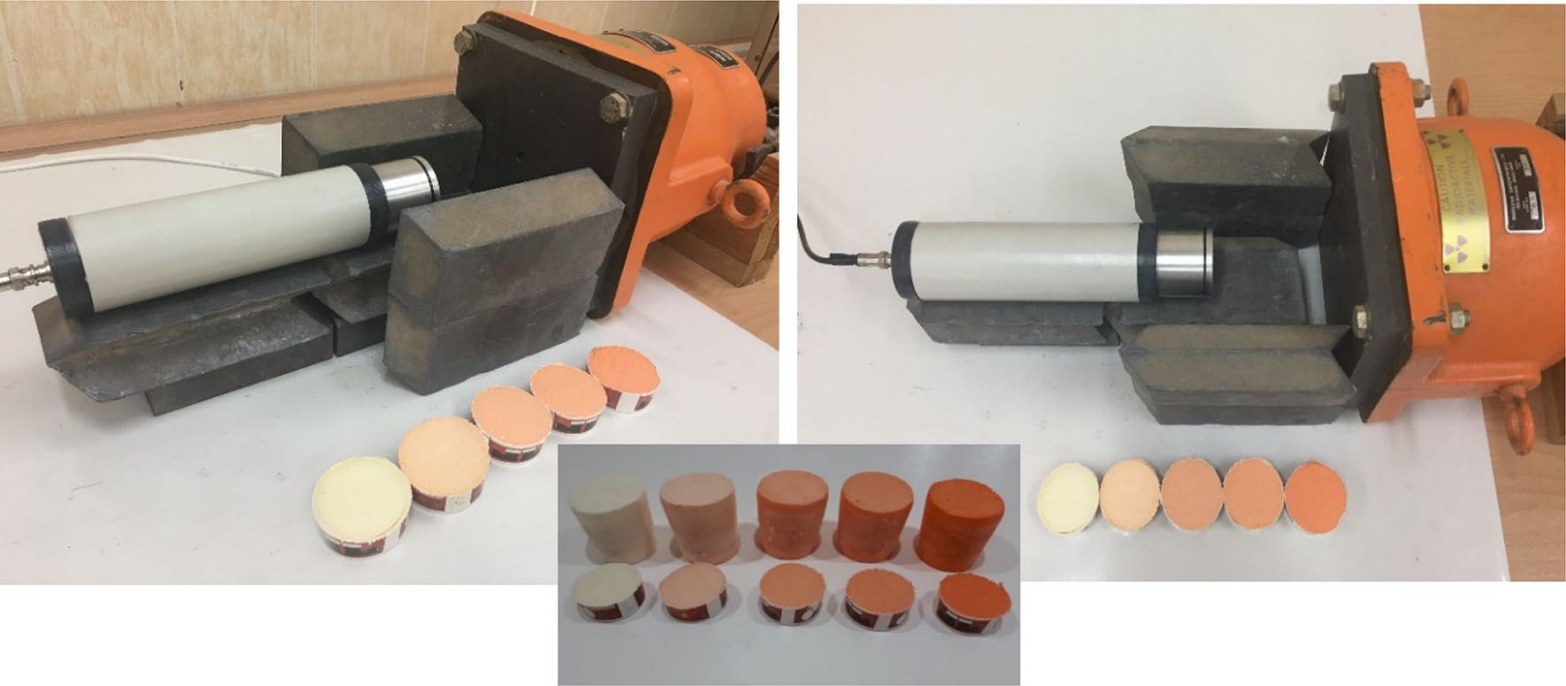
Experimental Set up.

This set-up was arranged as similarly as possible to the simulation set-up. Figure 5 presents the experimental set-up of this study.

## Results and discussion

**Figure 6:**
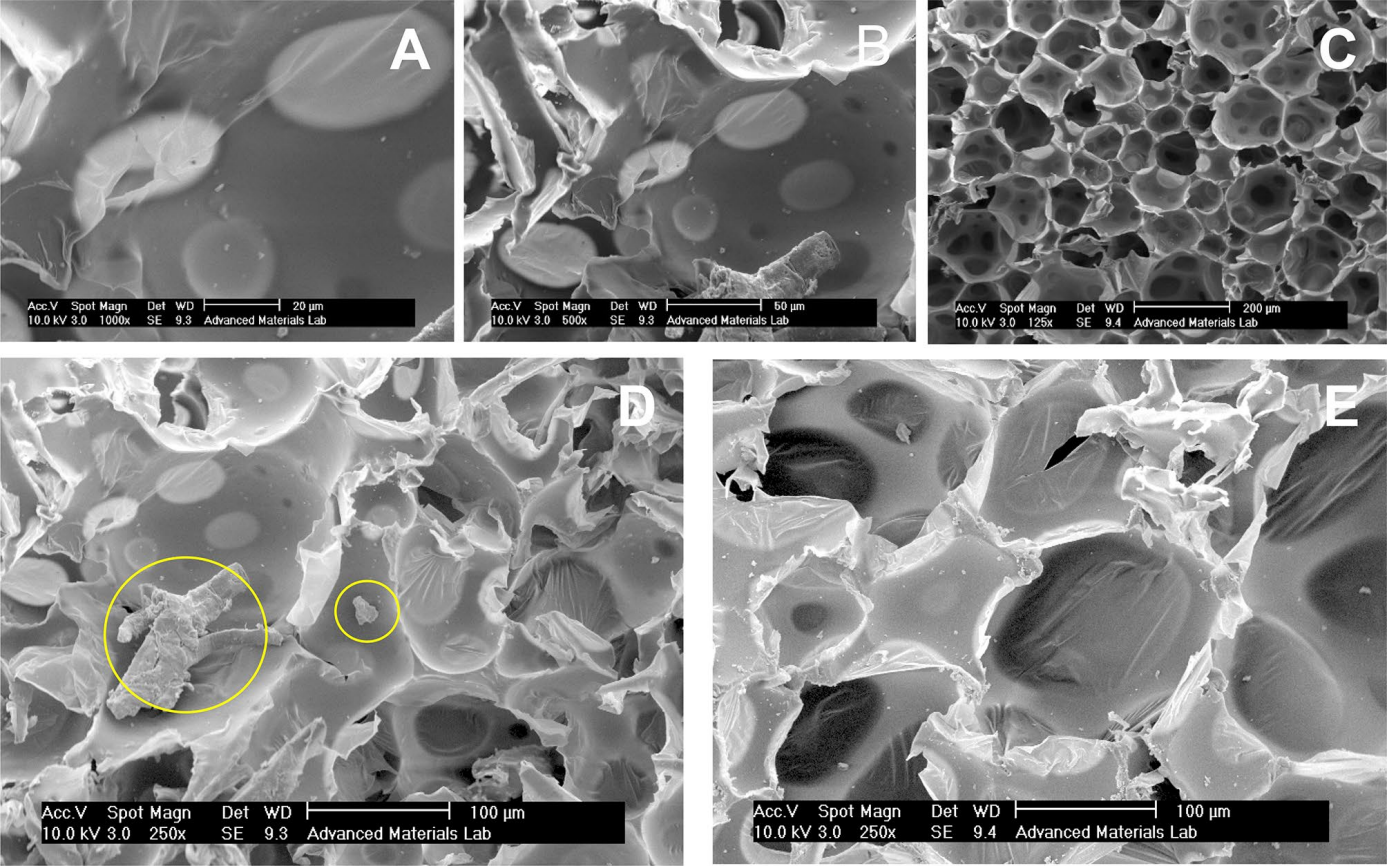
SEM results for generated samples in different scales.

The 5 samples, including one pure PUF, were available for further measurements. The changes in weight fraction led to several samples, reported in Table 1, with 0.2%, 0.5%, 1%, and 4% of PbO doped in polyurethane material during synthesis. After constructing the samples, the SEM and XRD analysis investigated. Figure 6 reports the SEM results for these samples. A, B and C panels depict the SEM of the polyurethane material plus PbO on scale of 20, 50 and 200 µm, respectively. In addition, panels D and E compare SEMs in scale of 100 µm in which D is polyurethane plus PbO and E represents pure polyurethane.

**Figure 7:**
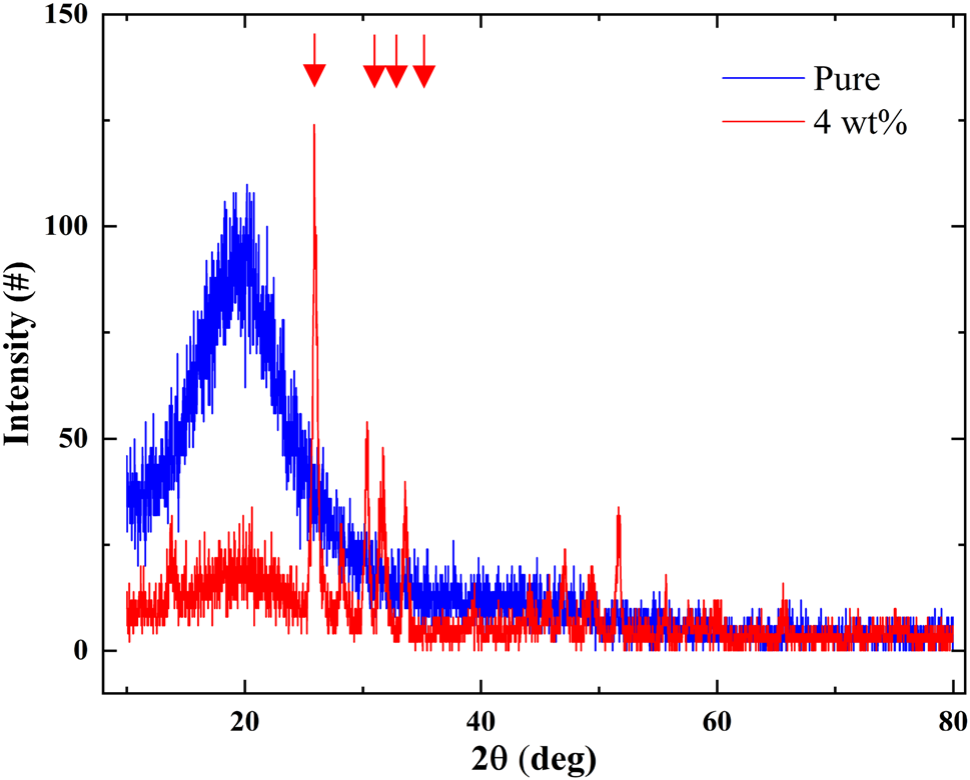
XRD spectra for pure and dopped sample.

The SEM results reported a visible PbO vision which was required. The doped PbOs are obvious in panel D. However, PbO was heavy and had a small fraction of the material to reduce the weight of the component. In addition to achieving the desired SEM results, the samples were tested via XRD to yield more precise results. The XRD results for the shield are reported in Fig. 7.

Here, the blue curve represents the pure PUF material, and the red curve is the PbO doped sample. The PbO peaks appearing in XRD curve proved the accuracy of the SEM results.

**Figure 8:**
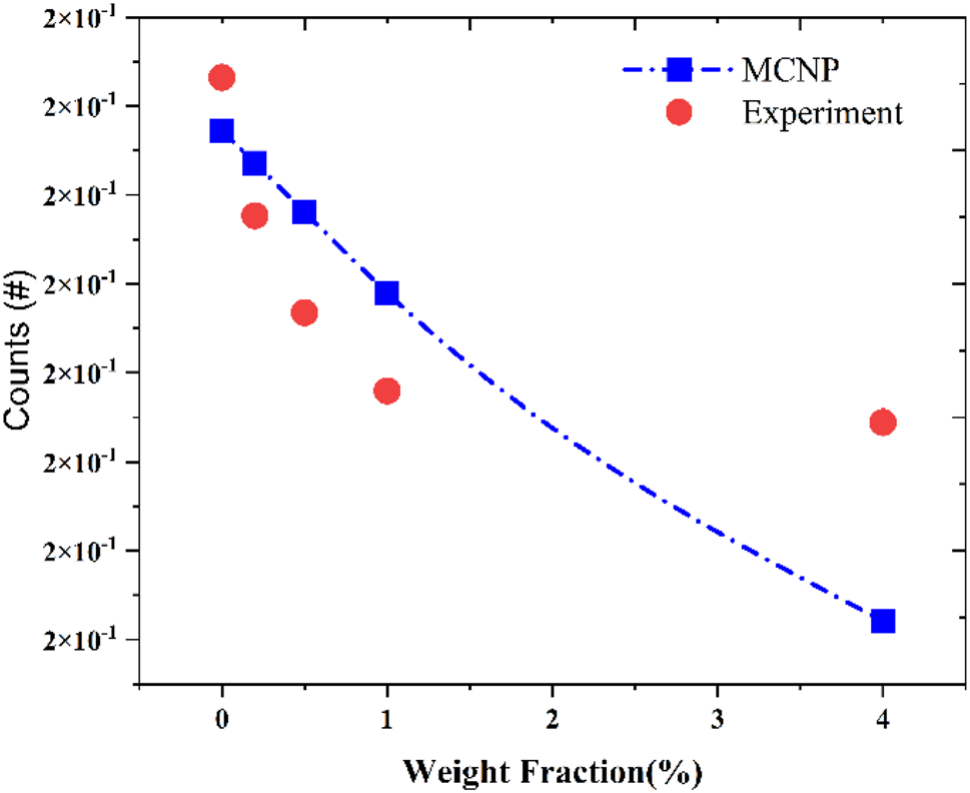
Number of counts for simulation and experiment.

Gamma particle detection was measured in the detector’s interring (see Fig. 1), and the calculations were performed with the obtained results in MCNP. Error bars used in the figure were very small (~ 10^–6^ order). According to the simulation results, the gamma attenuation of the considered shield increased by adding a heavy material such as PbO. Figure 8 reports the detected counts in simulation and experiment.

The results reported in Fig. 8 are obtained with tally F1 in the MCNPX. Source activity is considered and both results are normalized. The deviation between simulation and experiment, considering F4 tally and source intensity for the same sample, was about four percent. This deviation can be the result of some human and instrument mistakes in experiment. However, both methods had agreement in reducing the gamma counts with acceptable deviation.

**Figure 9:**
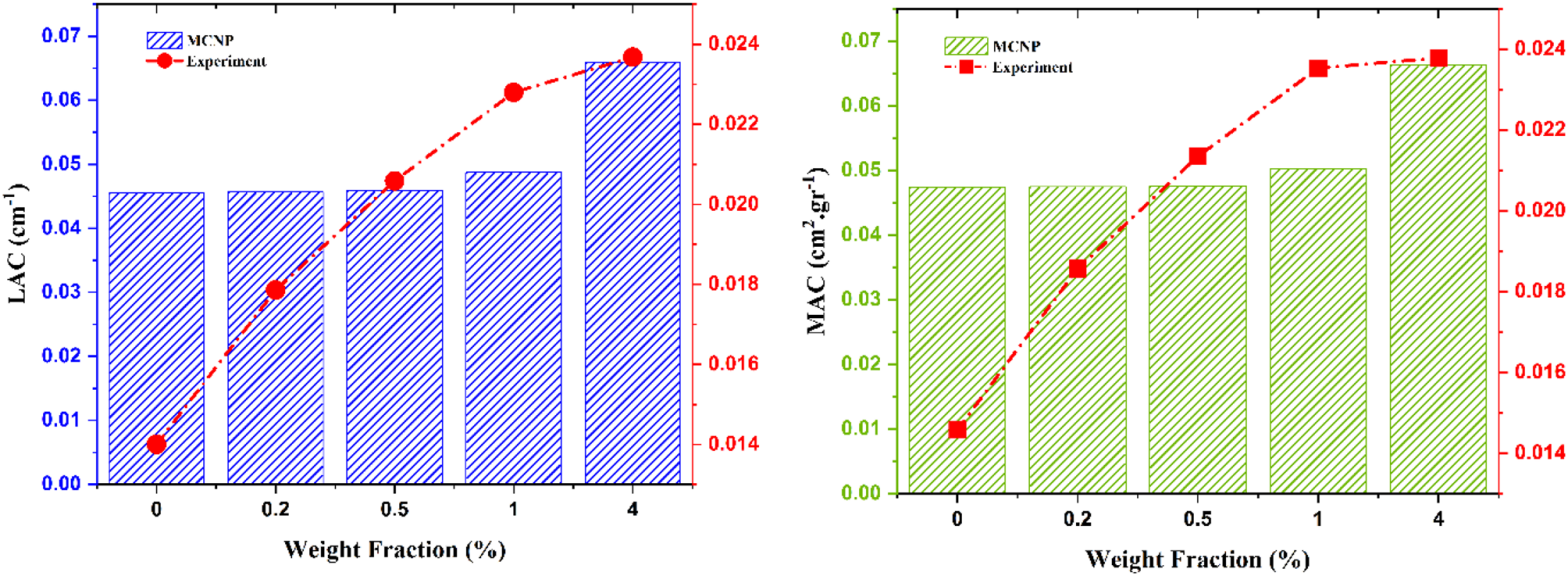
Linear attenuation coefficient and mass attenuation coefficient for experiment and simulation.

In addition to the count results, the samples were irradiated in simulation and experimental set-up (Figs. 1 and 5) in manner that was as similar as possible to each other. Figure 9 displays the radiation shielding properties of the constructed shields, including linear attenuation coefficient (LAC), mass attenuation coefficient (MAC).

**Figure 10:**
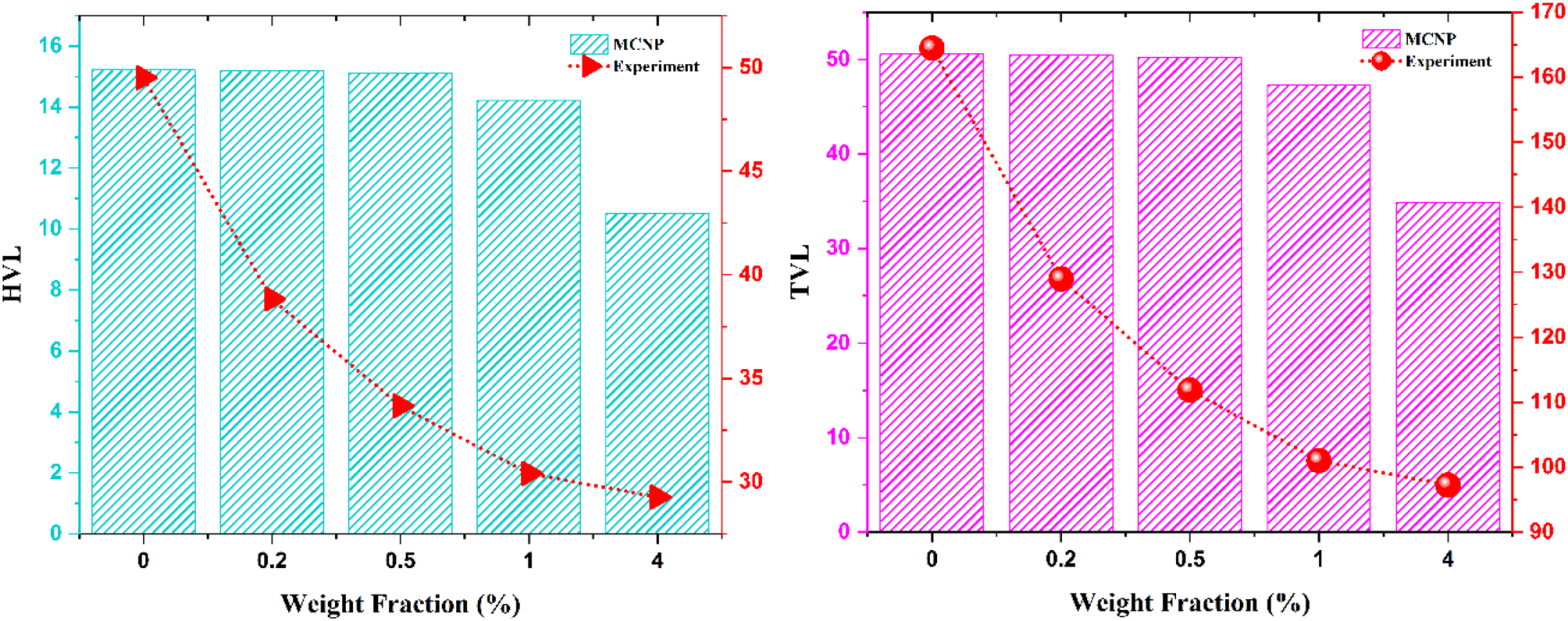
Half value layer and tenth value layer of the samples.

Moreover, utilizing Eqs. (2) and (3) the HVL and TVL of the samples are calculated and reported in Fig. 10.

The experiment was performed three times for each sample in 30 s, and the average was utilized in these calculations. All the samples successfully attenuated the radiation; however, as predicted, increasing the PbOs weight fraction caused more shielding properties. The sample with 4% PbO had more resistance against gamma radiation.

## Conclusion

Polyurethane foam (PUF) is one the most popular polymer nano composites worldwide due to its light-weight, available construction ingredients, the feasibility of using recycled material for its construction, and convenient construction in very short period. Nevertheless, it has not yet been properly investigated as a radiation shielding material.

This study constructed and explored PUF doped with PbO as a heavy component for gamma radiation shielding. MCNPX results showed a rise in the attenuation of gamma rays with the enhancement of PbO weight fraction and a decrease in gamma particle counts in the detector. The simulation results had a relative error less than 1%.

Based on the simulation outcomes and the acceptable results of MCNPX, the PU@PbO samples were constructed using the method mentioned in Section "Experiment", and the 5 obtained samples were radiated with ^137^Cs gamma source in 30 s for each sample. The results of the experiment and simulation are represented in Fig. 8.

The results in counts, mass attenuation coefficient (µ and µ/ρ), Half Value Layer (HVL), and Tenth Value Layer (TVL) in both simulation and experimental calculations were appropriate for shielding function and attenuating gamma radiation and were also consistent with each other. The SEM images showed a uniform dispersion of the inclusions into the polymer matrix, and the XRD analysis revealed the presence of PbO nanoparticles in the composite. At a higher percentage of PbO weight fractions, the value of attenuations of these nanocomposites against gamma radiation increased but it tended to saturate at much higher weight fractions.

The considered polyurethane-based gamma radiation shield is great for radiation protection, is lighter, has an easier construction, and exhibits convenient gamma shielding properties. It can be a suitable gamma shield for the nuclear safety of hospitals or other possible nuclear facilities and even in clothes in which it could be an appropriate substitute for traditional nuclear shielding materials.
